# Hemodiafiltration in Dialysis Practice: Global Adoption, Toxin Clearance, Inflammation Control, and Clinical Safety

**DOI:** 10.3390/toxins18080329

**Published:** 2026-07-28

**Authors:** Thomas Lang, Adam M. Zawada, Xiaoling Wang, Jochen G. Raimann, Manuela Stauss-Grabo, Nadja Grobe

**Affiliations:** 1 Clinical Research, Global Medical Office, Fresenius Medical Care Deutschland GmbH, 61352 Bad Homburg, Germany; thomas.lang@freseniusmedicalcare.com (T.L.); manuela.stauss-grabo1@freseniusmedicalcare.com (M.S.-G.); 2 Product Development, Fresenius Medical Care Deutschland GmbH, 66606 Sankt Wendel, Germany; adam.zawada@freseniusmedicalcare.com; 3 Clinical Research, Global Medical Office, Fresenius Medical Care, New York, NY 10065, USA; xiaoling.wang@rriny.com (X.W.);; 4 Katz School of Science and Health, Yeshiva University, New York, NY 10016, USA

**Keywords:** small-molecule solutes, middle-molecule solutes, protein-bound uremic toxins, inflammation, safety

## Abstract

Hemodiafiltration (HDF) combines diffusion and convection to enhance solute removal compared with hemodialysis (HD). While widely adopted in Europe and parts of Asia, uptake elsewhere remains limited due to regulatory, reimbursement, and infrastructural constraints. Recent randomized trials, large observational studies, and meta-analyses have renewed interest in HDF, particularly post-dilution HDF achieving convection volumes of ≥23 L/session, in which the most consistent clinical benefits have been observed. This review summarizes the mechanistic rationale, clinical effects, and safety profile of high-volume HDF. Compared with HD, HDF provides consistently superior removal of middle molecules, whereas clearance of protein-bound uremic toxins (PBUTs) remains constrained by albumin binding and compartmental redistribution, resulting in only modest or variable advantages. Emerging strategies aimed at releasing or capturing PBUTs may address this gap. Beyond solute removal, evidence suggests that HDF may lower micro-inflammation by enhanced clearance of inflammatory mediators and reduced immune activation. When delivered with adequate infrastructure, monitoring, modern equipment, ultrapure fluids, trained staff, appropriate vascular access, and anticoagulation, high-volume HDF has a favorable safety profile and is associated with fewer intradialytic hypotension (IDH) events and improved treatment tolerability. Available evidence has not identified a consistent excess risk of device- or infection-related complications, although comparative data remain limited. Overall, current evidence supports clinically relevant advantages of high-volume HDF under optimal treatment conditions. However, important challenges remain, particularly the limited removal of PBUTs, underscoring the need for further innovation in uremic toxin management.

## 1. Introduction

HDF is emerging as an important modality in chronic dialysis treatment worldwide; though its adoption varies widely across the globe [1]. In Europe and Asia, its use rapidly expanded supported by national guidelines and increasing familiarity with the modality. Furthermore, HDF is gaining momentum in developing countries, reflecting growing clinical interest and efforts to integrate the modality despite economic and technical constraints [2].

In contrast, adoption of HDF in the United States remains minimal as of 2026. The publication of the CONVINCE trial [3], followed by large cohort studies of adults treated across Europe, the Middle East, and Africa [4,5], renewed the global interest and prompted pilot programs and quality improvement initiatives aimed at evaluating feasibility, safety, and clinical outcomes in USA dialysis clinics [6].

This review summarizes current evidence regarding the adoption, mechanistic rationale, efficacy, safety, and implementation of HDF, with the aim of highlighting its potential benefits and informing its broader integration into clinical practice.

## 2. Global Adoption of HDF

The globally diverse adoption of HDF is shaped by a combination of clinical, technical, economic, and health-system factors.

Clinically, randomized and pooled analyses, e.g., ESHOL [7] and CONVINCE [3], along with recent meta-analyses [8,9], show that HDF reduces all-cause mortality versus conventional HD, particularly when higher convection volumes (≥23 L/session in post-dilution) are achieved [10]. This evidence has encouraged broader uptake in regions capable of operationalizing high-volume HDF in routine practice. Large real-world datasets, including national registries such as the Renal Epidemiology and Information Network (REIN) in France [11], the Australia and New Zealand Dialysis and Transplant Registry [12], and the Japanese Society for Dialysis Therapy Renal Data registry [13], further reinforce these survival signals and highlight consistent benefits across diverse patient populations.

Technically, HDF requires operating an HDF-capable machine, maintaining a validated ultrapure water system, and establishing workflows that sustain high blood-flow rates and safe filtration fractions. Recent optimization studies show that modern automation, such as tailoring the dialysate-to-blood flow (Qd/Qb) ratio to approximately 1.2 and using automated substitution control, can maintain high convective efficiency without increasing dialysate consumption [14]. These advances help lower the technical hurdles and resource concerns that limit HDF adoption.

Economically, upfront investments in HDF-enabled platforms, as well as potentially higher operating costs, remain important constraints for many dialysis providers. Where reimbursement structures recognize clinical value, adoption tends to accelerate. In resource-limited settings, clinicians have pursued lower-cost, resource-adapted approaches (e.g., simplified dilution schemes) to introduce convective therapy within budgetary constraints [2], underscoring financing and affordability as a primary determinant of diffusion.

Health-system and regulatory factors also play a critical role. Clear guidance and regulatory pathways catalyze implementation (e.g., European consensus and national recommendations), whereas delayed regulatory clearance has slowed adoption in some markets such as the United States. However, uptake is influenced by a broader set of factors beyond regulation alone. Reimbursement policies, the costs of upgrading and maintaining ultrapure water systems, investment in HDF-capable equipment, staffing requirements, workflow complexity, and the need for training and ongoing quality assurance may all limit implementation. Historically, the absence of strong incentives to convert from conventional HD to high-volume HDF has further contributed to heterogeneous adoption across healthcare systems. Importantly, where large provider networks and registries, such as REIN, Europe, Middle East, and Africa (EMEA) NephroCare, and European Clinical Database (EuCliD), support standardized protocols and outcome benchmarking, HDF transitions more readily from pilots to routine practice [11,15,16,17].

The recent publication of the CONVINCE trial [3,18], together with the 2025 ERA EuDial Consensus Statement [19], is likely to further influence global adoption of HDF by providing additional support for the use of high-volume HDF in appropriately delivered treatment settings. However, successful implementation remains dependent on adequate infrastructure, treatment quality, reliable vascular access, ultrapure water, and local expertise, which are likely to remain key determinants of its worldwide uptake.

## 3. Uremic Retention Solute Removal with HDF

The accumulation of uremic retention solutes contributes significantly to the high morbidity and mortality associated with end-stage kidney disease (ESKD). The effective removal varies considerably according to physicochemical properties and the dialysis modality employed. Low-flux HD relies almost exclusively on diffusive transport, whereas high-flux HD uses more permeable membranes that allow limited convective clearance of larger solutes [20]. High-volume HDF combines diffusion with substantial convection (≥23 L/session in post-dilution) by incorporating large-volume ultrafiltration and simultaneous reinfusion of substitution [21,22].

### 3.1. Role of Convection for Clearance of Small Water-Soluble Uremic Retention Solutes

Uremic retention solutes are traditionally categorized into three groups: small water-soluble compounds, middle molecules, and PBUTs [23]. Small water-soluble compounds, such as urea and creatinine, are efficiently cleared by diffusion. The additional convective component in HDF may provide marginal improvements to the clearance, but these gains are generally clinically less relevant, as adequacy targets (e.g., Kt/V) can be achieved with high-flux HD [24]. Phosphate, although a small solute, may behave differently due to its predominantly intracellular distribution and post-dialysis rebound kinetics. Studies suggest HDF to achieve increased phosphate removal compared to high-flux HD (estimates state up to 15% higher removal); however, if this translates into better long-term serum phosphate control warrants focused studies [19,25].

### 3.2. Removal of Middle-Molecule Uremic Toxins

Middle-molecule uremic toxins are generally defined as solutes with molecular weights between 500 Da and 60 kDa [23]. In contrast to small water-soluble compounds, the removal of middle molecules is most strongly affected by convection, and more specifically depends on membrane pore size. Naturally, HDF consistently achieves superior clearance compared to HD, particularly in post-dilution with achieved convective volumes of ≥23 L/session. As the most often referenced example, β2-microglobulin (β2M, 11.8 kDa) shows markedly higher reduction ratios (~10–20%) with HDF compared with high-flux HD [26,27,28]. HDF also enhances the removal of other clinically relevant middle molecules, including free light chains (κ-FLC, λ-FLC), and cytokines such as interleukin 6 (IL-6) and tumor necrosis factor α (TNF-α) [29,30].

Expanded hemodialysis (HDx), using medium cut-off (MCO) membranes, has emerged as an alternative approach to enhance middle-molecule removal through membrane design rather than high-volume convection and substitution fluid. Comparative studies have consistently shown that HDx achieves substantially greater single-session removal of middle molecules than conventional high-flux HD, including β2M, FLCs, myoglobin, and other larger uremic solutes [31,32]. However, direct comparisons between HDx and high-volume HDF have generally demonstrated broadly comparable middle-molecule removal [33,34,35], although some studies have reported greater clearance with high-volume HDF for selected middle molecules, including β2M and FLCs [32,36]. Overall, current evidence indicates that both HDx and high-volume HDF substantially improve middle-molecule clearance compared with conventional high-flux HD, but does not establish the superiority of either modality across the spectrum of middle molecules. Accordingly, HDx and high-volume HDF may be viewed as complementary approaches that achieve enhanced removal of middle molecules through different mechanisms [37]. Nevertheless, differences in single-session removal metrics should be interpreted cautiously, as enhanced intradialytic removal does not necessarily translate into lower long-term pre-dialysis concentrations or improved clinical outcomes.

### 3.3. Elimination of PBUTs

PBUTs represent the most challenging category of uremic retention solutes due to their extensive and reversible binding to plasma proteins, predominantly albumin. Only the unbound fraction is available for dialytic removal, which fundamentally limits clearance irrespective of dialysis modality.

PBUTs are not irreversibly attached to albumin but are reversibly bound through non-covalent interactions, including hydrophobic forces, electrostatic interactions, and hydrogen bonding [23,38]. Their distribution between bound and free fractions is governed by a dynamic dissociation equilibrium:

Albumin + Toxin ⇌ Albumin–Toxin

**Figure 1:**
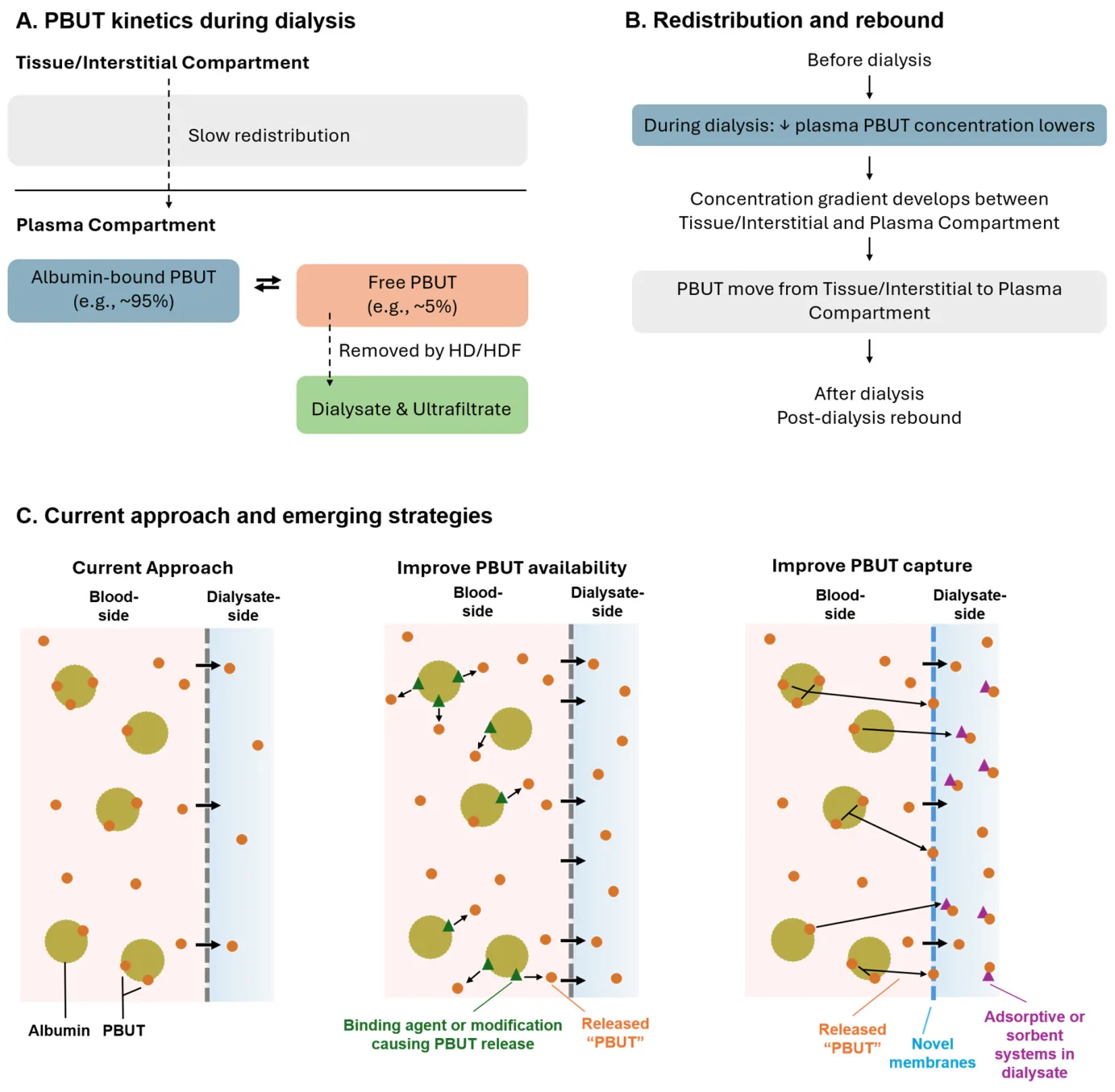
Key mechanisms limiting PBUT removal during dialysis and potential strategies to overcome them. (**A**) Equilibrium shifts between albumin-bound and free PBUT fractions during dialysis. Only the free fraction can cross the dialyzer membrane and be removed, resulting in continuous dissociation of PBUTs from albumin to re-establish equilibrium. (**B**) Intercompartmental redistribution of PBUTs between tissue and vascular compartments during and after dialysis, contributing to post-dialysis rebound in plasma concentrations. (**C**) Emerging strategies to enhance PBUT removal by increasing PBUT availability (e.g., binding-displacement approaches) and improving PBUT capture (e.g., adsorptive and novel membrane-based technologies).

This equilibrium is characterized by a dissociation constant (K_D) and reflects continuous exchange between both fractions [39]. During dialysis, removal of the free fraction lowers its plasma concentration, thereby shifting the equilibrium toward dissociation according to the law of mass action [39]. Additional toxins are released from albumin and become available for clearance. However, because albumin concentration is high and binding affinity is substantial, protein binding remains the principal limiting factor for PBUT elimination [39]. This thermodynamic constraint applies equally to HD and HDF (Figure 1A) [39,40].

Comparative clinical and crossover studies consistently demonstrate that PBUTs such as indoxyl sulfate (IS) and p-cresyl sulfate (pCS) exhibit only transient or modest improvements in removal with HDF compared with high-flux HD [41]. Different convective volumes appear to contribute to these gains. For example, Duval-Sabatier et al. [42] reported enhanced pCS removal at post-dilution HDF with substitution volumes of approximately 17 L, and Lima et al. observed greater reductions in circulating IS and pCS with post-dilution HDF delivering convection volumes of >27.5 L [43]. However, the clinical significance of these quantitative differences remains uncertain and requires further studies.

### 3.4. Impact of Compartmental Distribution and Rebound Kinetics on Uremic Toxin/PBUT Removal

Beyond membrane properties and transport mechanisms, dialysis efficacy is strongly influenced by the distribution of solutes across body compartments. Many uremic toxins are not limited to the intravascular space but are also present in interstitial and intracellular compartments. Since dialysis mainly clears the plasma pool, this heterogeneous distribution inherently limits total solute removal [44]. During dialysis, rapid reduction in plasma concentrations creates gradients that drive solute movement from extra- to intravascular compartments (Figure 1A). However, intercompartmental transfer is often slower than extracorporeal clearance. After dialysis ends, continued redistribution leads to a rise in plasma levels (“rebound”) [45,46,47]. The magnitude of this rebound depends on the volume of distribution, transfer rates, and intracellular storage. Solutes with large distribution volumes and slow equilibration are particularly affected [48]. Phosphate is a classic example: despite efficient removal during dialysis, serum levels increase again due to redistribution from intracellular stores (“phosphate rebound”). Because dialysis mainly clears the extracellular compartment, this limits net phosphate removal and complicates long-term control [49,50,51]. Similar mechanisms apply to middle molecules and, to a lesser extent, PBUTs [39]. While PBUT removal is primarily constrained by protein binding, their extravascular distribution adds another kinetic limitation (Figure 1B). Even when the free fraction is cleared, delayed redistribution can reduce net removal. In high-volume HDF, enhanced convective transport increases intravascular clearance but does not accelerate intercompartmental transfer. Therefore, higher extracorporeal clearance does not necessarily result in proportional increases in total removal from the body. Overall, dialysis efficacy reflects the interplay between clearance, intercompartmental mass transfer, and treatment time and frequency. Approaches that better account for slow redistribution, such as longer or more frequent dialysis, may improve net solute removal more effectively than simply increasing instantaneous clearance.

In addition to convection-based strategies, several emerging approaches aim to enhance PBUTs clearance by increasing the availability of the free PBUT fraction, including the use of chemical displacers, high ionic strength, or pH modulation to interfere with albumin binding (Figure 1C) [52]. Moreover, adsorptive techniques and sorbent systems, or novel mixed-matrix membranes, along with experimental bioartificial kidney systems, are being explored to further augment PBUT removal, although substantial challenges remain before these methods can be applied clinically [52].

In summary, the efficiency of uremic toxin removal is determined not only by dialysis modality but also by the physicochemical properties and kinetic behavior of individual solutes. While diffusion-based therapies adequately clear small water-soluble compounds, convection, particularly as implemented in high-volume HDF, provides clear advantages for the removal of middle molecules. In contrast, PBUTs remain largely refractory to dialytic elimination because of strong protein binding and unfavorable distribution kinetics, which limits the incremental benefits of increased convective clearance. Furthermore, multicompartmental distribution and post-dialysis rebound substantially constrain net solute removal across all toxin classes, underscoring that higher instantaneous clearance does not necessarily result in greater total elimination. Future strategies to improve uremic toxin control will therefore require approaches that address protein binding, compartmental kinetics, and treatment time and frequency, rather than relying solely on enhanced extracorporeal clearance.

## 4. Inflammation and HDF

**Figure 2:**
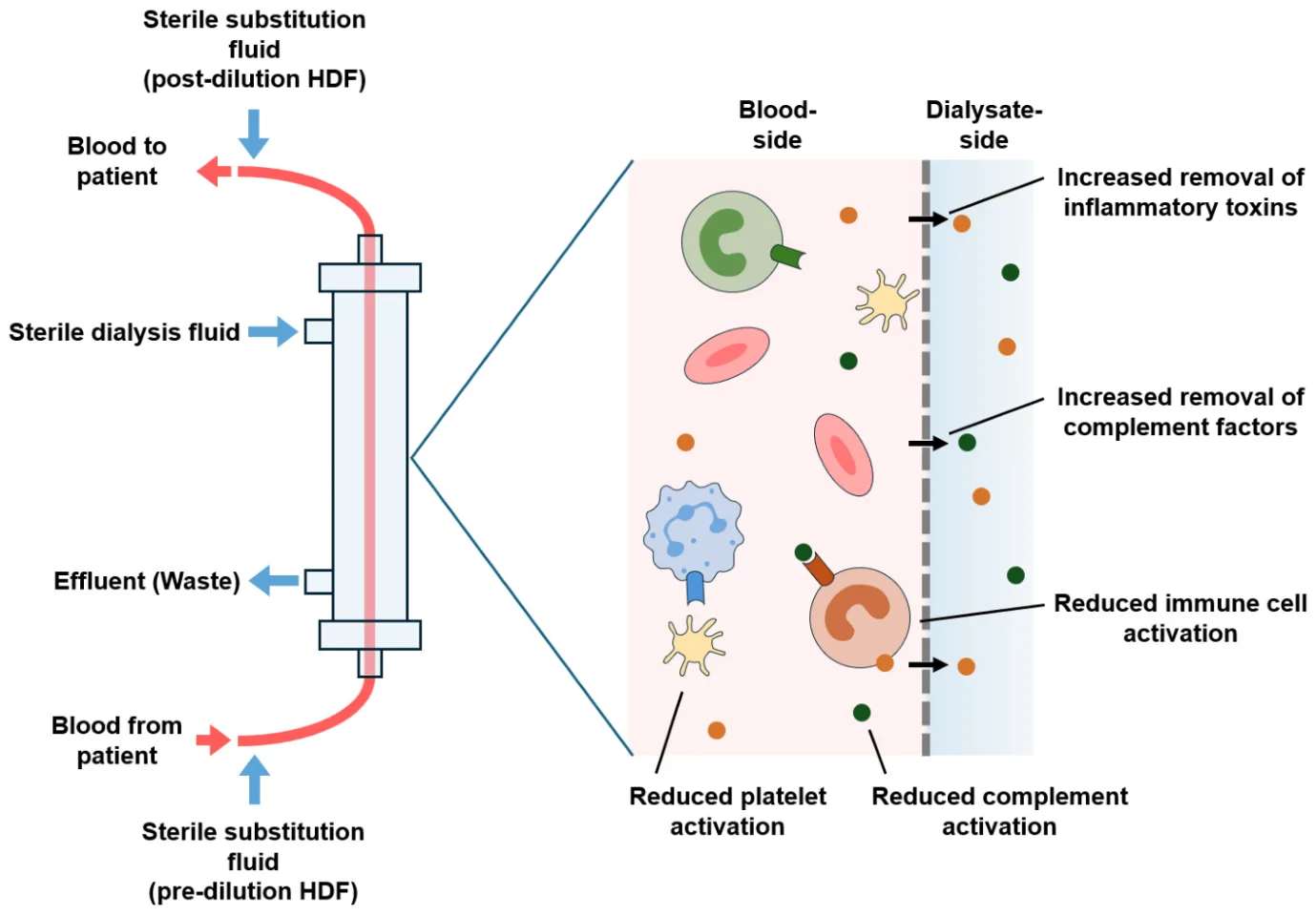
Proposed effects of high-volume HDF on inflammation. Conceptual illustration of inflammatory biomarkers and pathways that have been reported to be influenced by HDF, including circulating cytokines (e.g., IL-6, TNF-α), complement factors (e.g., complement factor D) and immune cell activation (e.g., proinflammatory CD16+ monocytes).

Chronic inflammation is a major burden for dialysis patients and a central contributor to the high morbidity and mortality rates [53,54,55,56]. While strong evidence linking improved patient survival to an improved inflammatory status in patients treated with high-volume HDF is lacking, biomarker studies indicate that HDF may be associated with a lower inflammatory burden. Proposed mechanisms include enhanced elimination of inflammatory cytokines and complement factors, as well as reduced immune cell activation as compared to standard HD treatments (Figure 2 and Table 1).

### 4.1. Reduction in Inflammatory Toxins by HDF

Uremia is characterized by an accumulation of inflammatory mediators due to the loss of renal clearance and by increased generation of these mediators through repeated immune activation during dialysis procedures. As highlighted in this section, high-volume HDF may reduce this inflammatory stress during each session, resulting in sustained benefits for the patients.

Elevated levels of cytokines such as IL-6, TNF-α and interleukin 1β (IL-1β) are associated with lower patient survival, and increased clearance of these compounds has been suggested as a potential contributor to the survival benefits observed with HDF [57]. When comparing the impact of treatment modality on the inflammatory status, results from the randomized controlled CONTRAST (CONvective TRAnsport STudy) trial among 405 patients demonstrated that—over three years of follow-up—concentrations of C-reactive protein (CRP) and IL-6 increased in patients treated with low-flux HD whereas the levels remained stable in patients treated with HDF, with a delivered convection volume of <23 L/session [58]. Similarly, the prospective observational RISCAVID (RISchio CArdiovascolare nei pazienti afferenti all’ Area Vasta In Dialisi) study among 757 patients and 30 months of follow-up found significantly lower IL-6 levels in patients treated with high-volume HDF as compared to HD [59]. Other markers of inflammation and oxidative stress, including TNF-α, IL-18, oxidized LDL, advanced glycation end-products and reactive oxygen metabolites, have also been investigated in the context of HDF and generally show lower levels with this modality [60,61,62,63].

### 4.2. Reduction in Complement Activation by HDF

Activation of the complement system is a common undesired effect during HD and contributes substantially to the inflammatory burden of patients [64,65,66,67,68,69]. Contact of blood with the artificial surfaces of the extracorporeal circuit primarily triggers the alternative pathway of the complement cascade, leading to the generation of complement components such as complement factor D, C3a, C5a and the terminal cytolytic membrane attack complex C5b-9 [64,65,66,67,68,69]. These factors promote inflammation by further activating leukocytes, platelets and the coagulation process.

HDF may be beneficial by improving the removal of complement factors. In a prospective, randomized clinical trial, Ward et al. compared the removal rate of complement factor D between online post-dilution HDF and high-flux HD among 45 patients over a follow-up time of 12 months [70]. HDF achieved significantly higher removal rates than HD and was associated with a significantly stronger decrease in pre-treatment complement factor D plasma concentrations over time [70]. These findings were confirmed in a recent crossover clinical trial among 20 patients who were treated with two different dialyzer types during HDF and HD, respectively [71]. For both dialyzer types, the HDF treatments were associated with higher removal rates of complement factor D as compared to HD treatments.

Besides better removal of complement factors, high-volume HDF may also attenuate complement activation by using ultrapure fluids and higher ultrafiltration rates, thereby minimizing back-filtration and potential endotoxin transfer which may occur with standard high-flux HD [30].

### 4.3. Reduction in Immune Cell Activation by HDF

Patients with ESKD exhibit profound immune dysregulation, including both functional impairments and shifts in immune cell populations [72,73,74]. The repeated contact of blood cells with artificial surfaces during dialysis contributes to this immune dysregulation. Within minutes of treatment initiation, leukocyte counts in the peripheral blood drop due to transient adherence to the patient’s vasculature [73,74]. Among these cells, a subpopulation of CD16-expressing monocytes (intermediate CD14++CD16+ and non-classical CD14+CD16++ monocytes) show the most pronounced decline during dialysis treatments. Importantly, cell counts of CD14++CD16+ monocytes are highly elevated in dialysis patients and independently predict cardiovascular events and mortality [75]. These cells have a high proinflammatory potential, producing ROS, IL-1β and TNF-α, and their differentiation is promoted by uremic conditions [76,77,78].

Therefore, strategies to reduce these cells, especially in dialysis patients, may help to mitigate the excessive cardiovascular risk in ESKD patients. In a prospective crossover study in 31 patients alternating between high-flux HD and high-volume HDF over four successive 4-month periods, the number of CD16-expressing monocytes was significantly lower during the HDF periods compared to the HD periods [79]. The lower cell counts of CD16-expressing monocytes were also accompanied by lower IL-6 and TNF-α levels. These results were confirmed in a clinical study with 15 dialysis patients who were switched from high-flux HD (baseline) to HDF and back to high-flux HD, both periods lasting 4 months each [80]. During the HDF period, the numbers of CD16-expressing monocytes were significantly lower than during baseline and the following HD period. Moreover, monocyte-derived dendritic cells showed lower activation during HDF as compared to HD in a longitudinal clinical study involving 31 dialysis patients, with each treatment modality applied over a four-week period [81].

**Table 1:** Overview of studies comparing inflammatory markers in HD vs. HDF.

Category	Study	Study Design	Patient Number	Follow-Up	Comparison	Biomarker	Study Results
Inflammatory toxins and oxidative stress	den Hoedt et al., 2014 [CONTRAST] [58]	Randomized clinical trial	405	3 years	Low-flux HD vs. online post-dilution HDF	CRP, IL-6	CRP and IL-6 increased in HD and remained stable in HDF
	Panichi et al., 2008 [RISCAVID] [59]	Prospective observational study	757	30 months	Standard bicarbonate HD vs. HDF with sterile bags vs. high-volume HDF	CRP, IL-6	IL-6 reduced in online HDF versus HD and HDF with bags
	Agbas et al., 2018 [60]	Prospective observational study	22	3 months per treatment modality	High-flux HD vs. online HDF	IL-6, IL-10, CRP, nitrotyrosine, AGEs, ox-LDL, ADMA, SDMA, TAC	CRP, ADMA, SDMA, AGEs, ox-LDL reduced in HDF, TAC increased in HDF
	Filiopoulos et al., 2008 [61]	Prospective observational study	9	9 months	Post-dilution HDF vs. baseline (HD)	TAC, d-ROMs, SOD, CRP, IL-6	d-ROMs, CRP decreased and TAC increased over time
	Fischer et al., 2021 [62]	Prospective observational study	144	12 months	HD vs. post-dilution HDF	IL-6, TNF-α, CRP	IL-6, TNF-α, CRP lower at baseline and after 12 months with HDF
	Kuo et al., 2008 [63]	Prospective observational study	17	6 months	Online post-dilution HDF vs. baseline (HD)	IL-6, IL-18, TNF-α, CRP	IL-18 and TNF-α decreased over time
Complement activation	Ward et al., 2000 [70]	Randomized controlled trial	45	12 months	Online post-dilution HDF vs. high-flux HD	Complement factor D	Higher removal rates and stronger decrease in pre-treatment complement factor D levels over time with HDF
	Elsayed et al., 2024 [71]	Prospective crossover study	20	4 weeks (2x HD and 2x HDF) with two-week washout period between sessions	Online post-dilution HDF vs. high-flux HD	Complement factor D	Higher removal rates of complement factor D with HDF
Immune cell activation	Carracedo et al., 2006 [79]	Prospective crossover study	31	16 months (4 × 4 months, following baseline)	Online HDF vs. high-flux HD	CD16-expressing monocytes	Lower number of CD16-expressing monocytes with HDF
	Ramirez et al., 2007 [80]	Prospective crossover study	15	8 months (4 months per modality, following baseline)	Online HDF vs. high-flux HD	CD16-expressing monocytes	Lower number of CD16-expressing monocytes with HDF
	Rama et al., 2016 [81]	Prospective longitudinal study	31	8 months (4 months per modality)	Online HDF vs. high-flux HD	Monocyte-derived dendritic cells	Lower activation of monocyte-derived dendritic cells with HDF

HD: hemodialysis, HDF: hemodiafiltration, CRP: C-reactive protein, IL: Interleukin, AGEs: advanced glycation end-products, oxLDL: oxidized low-density lipoprotein, ADMA: asymmetric dimethyl arginine, SDMA: symmetric dimethyl arginine, TAC: total antioxidant capacity, d-ROMs: reactive oxygen metabolites, SOD: superoxide dismutase, TNF-α: tumor necrosis factor α.

In summary, increasing evidence suggests that HDF may reduce the inflammatory burden of dialysis patients by improving the removal of inflammatory toxins, complement factors as well as attenuating immune cell activation compared to conventional HD. However, the available evidence is derived largely from observational studies focusing on biomarkers, and a causal link between these findings and improvements in hard clinical outcomes is still lacking.

## 5. Patient Safety with HDF

Dialysis patients are vulnerable to acute and chronic treatment-related complications, so any dialysis modality must demonstrate not only efficacy but also a sustainable safety profile. It is therefore crucial to understand where HDF provides clear safety benefits, where specific risks may arise, and how these can be mitigated. The following section examines safety aspects across acute and chronic complications, systemic and technical safeguards, device-related events, and patient experience (see also Table 2). Emphasis is placed on mechanistic explanations for the differences between the modalities and on practical implications for safe implementation.

### 5.1. Acute Risks

#### 5.1.1. IDH and Hemodynamic Stability

Episodes of IDH are common complications of HD [82] associated with reduced quality of life (QoL), increased risk of myocardial ischemia, cerebral hypoperfusion, higher mortality, and rehospitalization [30,83,84,85,86]. IDH is an objectively defined complication with distinct pathophysiology, clear prognostic importance, unlike other mostly patient-reported dialysis symptoms (e.g., cramps, nausea, headache, fatigue, itching). Conventional high-flux HD exhibits higher IDH rates than high-volume HDF, as seen in multiple studies such as ESHOL HOLLANT and Italian Convective [7,87,88,89,90,91,92]. This hemodynamic stability may be explained by the convective component of HDF, which supports plasma refill and reduces thermal stress [30,85,86,90,93].

#### 5.1.2. General Dialysis-Related Symptoms

Cramps, nausea, headaches, dizziness, pruritus, and post-dialysis fatigue reduce the QoL of patients and can impair adherence, such as early termination of treatment sessions [94,95,96,97,98]. Patient-reported outcome (PRO) data from CONVINCE and subsequent analyses demonstrate that patients treated with high-volume HDF report fewer intradialytic symptoms and shorter recovery times [99]. These differences may be explained by the underlying physiology: improved hemodynamic stability limits symptomatic hypotension and cramp-provoking ischemia; enhanced convective clearance reduces the burden associated with middle molecules (e.g., cytokines, β2M), such as malaise and fatigue [14,30,99]. Collectively, controlled trials and mechanistic reviews indicate that tolerability during and after treatments is superior with high-volume HDF than with standard HD treatments [89,99,100,101,102,103].

**Table 2:** Safety differences in HD vs. high-volume HDF.

Category	Safety Aspect	High-Volume HDF	Quantitative Evidence	Level of Evidence	Mechanistic Rationale/Comments	Key References
Acute Risks	IDH	↑ Less frequent	Symptomatic hypotension significantly lower (OR 0.46; 95% CI: 0.33–0.63, *p* < 0.001) [87] and Rate Ratio (0.72; 95% CI: 0.68–0.77; *p* < 0.001) [7]. Number of early-onset IDH episodes less frequent (0.32/session vs. 0.10/session; *p* = 0.001) [88]. Asymptomatic hypotension reduced (OR 0.87, 95% CI: 0.79–0.95, *p* = 0.002) [89]	RCTs	HDF improves plasma refill and reduces thermal load, leading to more stable hemodynamics.	[7,30,87,88,89]
	General dialysis-related symptoms	↑ Fewer symptoms, faster recovery	Higher satisfaction level with less cramps, itching, joint pain and stiffness, and post-dialysis fatigue (*p* < 0.001) [100]. Fewer episodes of muscle cramps (*p* = 0.03) [89]	RCTs	Enhanced middle-molecule clearance and better hemodynamic control may contribute to improved tolerability in HDF.	[30,89,99,100]
	Electrolyte shifts	↑ Higher removal	Serum phosphate reduced (β = −0.36 mg/dL, 95% CI: −0.65 to −0.06, *p* = 0.02 [104], 1.53 ± 0.53 mmol/L in HD vs. 1.42 ± 0.61 mmol/L in HDF, *p* < 0.001 [105]; 5.1 ± 1.0 mg/dL in HD vs. 4.0 ± 0.7 mg/dL in HDF; *p* < 0.0001 [106]	RCT/Observational studies	Convective transport increases phosphate clearance and may improve phosphate control; risk of hypophosphatemia requires monitoring.	[25,104,105,106,107]
Chronic Risks	Mortality	↑ Lower risk (with high convection volumes)	All-cause mortality significantly reduced: CONVINCE study (HR = 0.77; 95% CI: 0.65–0.93; *p* = 0.005) [3]; ESHOL study (HR = 0.70; 95% CI: 0.53–0.92; *p* = 0.01) [7]; CONTRAST study (exploratory high-volume/on-treatment analysis, HR = 0.62; 95% CI: 0.41–0.83; *p* = 0.01) [108]; Turkish OL-HDF study (subgroup >17.4 L/session, RR = 0.54, 95% CI: 0.31–0.93, *p* = 0.02) [109]. Infection-based mortality significantly reduced: CONVINCE study (including COVID-19, HR = 0.69; 95% CI: 0.49–0.96), [3]; ESHOL study (HR, 0.45; 95% CI: 0.21–0.96; *p* = 0.03) [7]. Cardiovascular mortality significantly reduced: ESHOL study (HR, 0.67; 95% CI: 0.44–1.02; *p* = 0.06) [7]; Turkish OL-HDF study (subgroup >17.4 L/session, RR = 0.29, 95% CI: 0.12–0.65; *p* = 0.003 [109]	RCTs/Post-hoc RCT analysis/IPD Meta-analysis	High-volume HDF reduces IDH, systemic inflammation, and clears cardiotoxic solutes, lowering mortality.	[3,7,9,10,30,108,109]
	β2M/amyloidosis	↑ Lower accumulation	Significant reduction in carpal tunnel syndrome surgery (RR = 0.58, 95% CI: 0.35 to 0.95, *p* = 0.03) [110]	RCTs	Superior clearance of β2M in HDF may reduce amyloidosis progression risk.	[110,111,112,113,114]
	Amino acid/peptide losses	Slightly increased losses	−	Mechanistic and clinical metabolic studies	Convective transport in HDF leads to mild amino acid and peptide losses, usually clinically insignificant but may be relevant in malnourished patients.	[115,116]
	Nutritional markers (nPNA)	↑ Higher protein intake	Normalized protein appearance increased (mean difference 0.26 g/kg/day, 95% CI: 0.05–0.47; *p* = 0.002) [117]	RCTs	Higher normalized protein appearance may reflect improved protein intake and maintenance of nutritional status during OL-HDF.	[117]
Dialysis Procedure-and Equipment-Related Adverse Events (ADEs)	Hemolysis	Rare in both modalities	−	Review-based clinical and mechanistic evidence	Mechanical stress or overheating can cause hemolysis; modern QA minimizes risk equally.	[118,119]
	Membrane leaching & toxin retention	Lower risk	−	Mechanistic evidence	HDF uses higher-quality membranes and online substitution fluid, reducing backfiltration and leaching risks.	[120]
	Pyrogenic reactions	Rare (ultrapure water) in both modalities	−	Observational and technical/process evidence	Modern ultrapure systems keep risk of microbial contamination very low in both modalities.	[119,121,122]
	Clotting & thrombogenicity	Slightly higher risk	−	Mechanistic and review-based clinical evidence	Higher filtration fractions increase clotting risk in HDF without optimized anticoagulation.	[123]
	Vascular access complications	Comparable in both modalities	−	Observational evidence/expert review	Access complications occur similarly; HDF’s more stable hemodynamics may protect vascular access over time.	[124,125]
	Mechanical & technical failures	Slightly increased technical requirements	−	Expert review/technical evidence	HDF requires modest additional machine function; failure risk is strongly minimized by preventive maintenance and staff training.	[20]
Patient Experience	Treatment tolerability & QoL	↑ Often improved	Significant improvement in cognitive function (Delta/Mean change = 2.95 points, 95% CI: 1.06–4.84, *p* < 0.05) and improvement in physical function, social interaction and pain interference) [99]. Higher satisfaction level with improvement in general mood, sexual performance and social activity (Mean ± SD 94 ± 9 vs. 28 ± 16, 57 ± 10 vs. 5 ± 5, 82 ± 9 vs. 15 ± 8, *p* < 0.0001, respectively) [100]	RCTs/Observational studies/Meta-analysis of RCTs	Patients report better tolerability, improved cognitive function, less fatigue, and improved recovery in HDF with high convection volumes.	[30,99,100,126,127]

Acronyms used: HD, hemodialysis; HDF, hemodiafiltration; OL, Online; IDH, intradialytic hypotension, nPNA, normalized Protein Nitrogen Appearance; QA, Quality assurance; OR, odds ratio; β2M, β2-microglobulin; dL, deciliter; *p*, *p*-value; HR, hazard ratio; RR, relative risk; CI, confidence interval; SD, standard deviation; QoL, quality of life; RCT, randomized controlled trial; IPD, individual participant data. Symbols indicate the direction of effect only where comparative clinical evidence is available: ↑ indicates a more favorable outcome with HDF. No directional symbol is assigned where comparative evidence is insufficient or primarily mechanistic, observational, or expert-based.

#### 5.1.3. Electrolyte Shifts and Osmotic Imbalance

Both modalities are effective in removing small solutes such as phosphate, sodium and potassium. While the convective component of HDF may enhance phosphate clearance beyond diffusion alone, sodium and potassium removal remains predominantly diffusion-driven, with no consistent additional benefit from convection [25,104,105,106,107,108,128,129,130]. Augmented phosphate clearance with high-volume HDF may be advantageous in patients with persistent hyperphosphatemia associated with the risk of vascular and valvular calcification, a common challenge in dialysis populations frequently requiring phosphate binder therapy. While higher intradialytic phosphate removal can lead to lower post-dialysis levels, clinically relevant sustained hypophosphatemia appears uncommon when dialysis prescription and nutritional status are appropriately monitored [86,131,132].

### 5.2. Chronic Risks

#### 5.2.1. Mortality

Multiple randomized trials, meta-analyses, and observational studies suggest an association between high-volume HDF and lower all-cause, infection-related, and cardiovascular mortality compared with high-flux HD [3,7,9,10,12,13,19,109,133,134,135,136,137]. Importantly, these survival benefits appear most apparent when sufficiently high post-dilution convection volumes are achieved. Although the most consistent survival advantages across randomized trials and meta-analyses have generally been observed at post-dilution convection volumes of approximately 23 L/session or higher, the Turkish OL-HDF Study suggested survival benefit already at lower achieved convection volumes (>17.4 L/session) [109]. However, studies with lower mean delivered convection volumes overall have otherwise shown less consistent or neutral primary mortality outcomes [89,108,109]. Notably, although the primary intention-to-treat analysis of the CONTRAST trial was neutral for all-cause mortality (HR 0.95; 95% CI 0.75–1.20), an exploratory on-treatment analysis suggested improved survival among patients achieving high convection volumes (HR 0.62; 95% CI 0.41–0.83) [84]. This distinction highlights the importance of differentiating prespecified randomized outcomes from post-hoc analyses when interpreting the mortality evidence.

Many underlying mechanistic causes could be attributed for this survival benefit, including fewer IDH episodes, lower inflammatory status (e.g., reduced chronic micro-inflammation, improved clearance of pro-inflammatory cytokines; see Section 4), improved phosphate handling, and removal of cardiotoxic solutes (e.g., middle molecules, AGEs) [138].

The CONVINCE trial achieved particularly high treatment delivery targets, with a mean convection volume of 25.3 L/session and the predefined 23 L target reached in the large majority of HDF sessions [3]. In addition, most participants had arteriovenous vascular access, reflecting a population well suited for achieving high convection volumes. These factors may have contributed to the observed mortality benefit and should be considered when extrapolating the findings to broader dialysis populations or centers where such treatment targets are less consistently achievable.

Interpretation of the mortality evidence should consider important differences among the available studies. Randomized controlled trials differed with respect to the comparator modality (low-flux versus high-flux HD), achieved convection volume, HDF dilution mode (predominantly post-dilution versus pre-dilution), patient characteristics including vascular access and residual kidney function, as well as primary endpoint definitions and follow-up duration [3,7,9,10,13,108,109]. Importantly, the strongest evidence originates from prespecified primary analyses of randomized controlled trials, whereas survival signals from post-hoc subgroup analyses and observational registry studies should be interpreted as supportive rather than confirmatory evidence [9,10,11,12,108]. Furthermore, achieving high convection volumes depends not only on the treatment modality itself but also on treatment feasibility, including adequate vascular access, sufficiently high blood-flow rates, patient tolerance, and center experience. Consequently, achieved convection volume may partly reflect patient selection and implementation factors in addition to treatment efficacy, which should be considered when interpreting the reported survival associations [3,9,10,108].

#### 5.2.2. β2M and Dialysis-Related Amyloidosis (DRA)

β2M accumulation underpins DRA with musculoskeletal morbidity and risk for carpal tunnel syndrome [111,113,139,140,141,142]. HD and HDF incompletely clear β2M, whereas convective transport with HDF improves removal substantially [110,114]. Longitudinal studies document lower β2M levels and reduced DRA progression with long-term HDF treatments, representing a safety advantage of this modality vs. conventional HD [112].

#### 5.2.3. Nutritional Status

One potential limitation of convection is the potential greater loss of amino acids and small peptides vs. HD [115,116]. In most patients with adequate intake, these losses are clinically mild; however, in fragility or protein-energy wasting, they can be meaningful [143]. Mitigation includes individualized convection volumes, dialysate potassium/phosphate tailoring, nutritional counseling, and supplementation or intradialytic nutrition. All of these mitigation approaches need to be evaluated and implemented when indicated. Close surveillance of serum albumin and protein catabolic rate is essential [86,144]. Although HDF may induce mild amino acid and peptide losses, these should not be equated with clinical nutritional outcomes. Amino acid losses, normalized protein appearance (nPNA), serum albumin, muscle mass, and protein-energy wasting represent distinct physiological and clinical parameters and should therefore be interpreted separately [115,116,117]. Compared with HD, HDF has been associated with higher normalized protein appearance and better preservation of muscle mass, suggesting maintained or improved dietary protein intake despite modest convective amino acid losses [117].

### 5.3. Dialysis Procedure- and Equipment-Related Adverse Events (ADEs)

For several procedure- and equipment-related adverse events discussed below, robust comparative RCT data between HDF and HD are unavailable. Accordingly, the absence of a reported excess risk should not be interpreted as evidence of equivalent safety; conclusions for these outcomes rely largely on mechanistic, observational, technical, or expert-review evidence and should be interpreted cautiously.

#### 5.3.1. Hemolysis

Clinically significant hemolysis during dialysis is rare with modern membranes and thermal control [118,145]. When it occurs, it is typically related to the extracorporeal dialysis circuit or procedure, including overheating of dialysate, excessive negative pressures in the arterial line, mechanical shear forces, or rarely defective dialyzers (e.g., manufacturing defects in fibers) [119]. Available evidence has not identified a specific increase in hemolysis risk with HDF; however, robust comparative RCT data are lacking [146].

#### 5.3.2. Membrane Leaching and Toxin Transfer

Early-generation cellulose membranes could leach low-molecular compounds and allowed backfiltration of trace contaminants. Modern synthetic membranes used in both HD and HDF substantially reduce membrane leaching and improve biocompatibility [147]. In high-volume HDF, the mandatory use of ultrapure dialysate for substitution fluid preparation further minimizes the risk of endotoxin transfer, comparative clinical outcome data remain limited [148].

#### 5.3.3. Pyrogenic Reactions

High-volume HDF reinfuses large volumes of substitution fluid; therefore, microbiological safety is of utmost importance [119]. Contemporary systems employing multi-stage reverse osmosis, endotoxin-retentive ultrafilters, and rigorous disinfection achieve ‘ultrapure’ fluids, rendering clinical pyrogenic reactions are rare under these conditions, although robust comparative RCT data versus HD are lacking [119,122]. Surveillance cultures, endotoxin assays, and process validation are integral to maintaining this safety margin [119,121,149,150].

#### 5.3.4. Clotting and Thrombogenicity

Clotting within the dialyzer and extracorporeal circuit remains an important concern, as it compromises treatment adequacy and increases blood loss [151,152]. In HD, clotting risk is generally low with adequate anticoagulation [153]. However, in HDF, especially in high-volume HDF, the use of high convection volumes and higher filtration fractions may increase hemoconcentration within the filter, creating a more thrombogenic environment [152]. Careful adjustment of filtration fraction, modality, and individualized anticoagulation may mitigate this risk; however, robust comparative data on thrombogenic events between high-volume HDF and high-flux HD are limited [151].

#### 5.3.5. Vascular Access Complications

Stenosis, thrombosis, and catheter-related bloodstream infection rates are broadly similar between modalities because access type (e.g., arteriovenous fistulas, grafts, or central venous catheters) and cannulation technique dominate risk [124,154]. By reducing IDH and access hypoperfusion, HDF may preserve access patency, but definitive randomized evidence is lacking [125].

#### 5.3.6. Mechanical and Technical Failures

Complexity in HDF (substitution pumps, ultrafilter integrity tests, quality monitors) introduces more alarm opportunities; however, with preventive maintenance, competency training, and incident learning, clinically meaningful failures are rare [20]. Although HDF requires additional components such as substitution pumps, modern systems with automated substitution-fluid volume control systems have streamlined the process to make it as straightforward as HD in daily practice. Comparative clinical study data on technical failure rates between HDF and HD are not available.

Overall, available evidence has not identified a consistent signal of increased procedure-, device-, or infection-related risk with modern HDF compared with HD. However, for several specific outcomes, including hemolysis, pyrogenic reactions, thrombogenicity, vascular access complications, and technical failures, robust comparative RCT data are lacking. Therefore, current findings should be interpreted as an absence of a demonstrated excess risk rather than definitive evidence of equivalent safety.

### 5.4. Patient Experience

#### Treatment Tolerability and QoL

From the patient’s perspective, QoL, functional capacity, and symptom burden are essential indicators of dialysis adequacy. Fatigue, post-dialysis recovery time, sleep disturbances, and cognitive dysfunction are frequently reported by HD patients [99,155,156,157,158]. Randomized controlled trials and patient-reported outcome studies have shown that patients treated with HDF experience shorter recovery times, less fatigue and improved cognitive and physical functioning [3,30,99,100,126,159,160]. No effects were documented regarding sleep duration [161]. Mechanistically, these improvements may be attributed to enhanced clearance of middle molecules (e.g., cytokines, β2M, neurotoxins), which are linked to fatigue, malaise and cognitive defects, as well as more stable hemodynamics during HDF sessions [30,127].

In summary, investigating safety by the respective domains such as acute vs. chronic, systemic vs. technical, device-related, and experiential, a coherent pattern emerges. High-volume HDF provides superior hemodynamic stability (fewer IDH events), lower inflammatory burden, better clearance of middle molecules and phosphate, and lower β2M accumulation. All changes may plausibly contribute to the cardiovascular and survival advantages observed in randomized trials and meta-analyses [7,8,9,10,11,12,13,19,30]. Against these benefits stand manageable trade-offs: modest additional operational requirements, the need for ultrapure water and online substitution, and vigilant anticoagulation to prevent extracorporeal circuit clotting [30]. Importantly, these trade-offs have not been associated with a consistent increase in infection-related or serious device-related complications in modern programs adhering to quality standards. For centers equipped with HDF-capable platforms and robust water treatment, prioritizing high convection volumes while individualizing electrolytes and nutrition appears to optimize the safety/efficacy balance. Future work should refine patient selection (e.g., extreme cardiovascular risk, refractory hyperphosphatemia, high inflammatory burden), define pragmatic anticoagulation algorithms tailored to filtration fraction, and evaluate implementation strategies that lower the logistical barriers to HDF adoption—particularly in regions where HD remains dominant [30].

## 6. Limitations

We aimed to characterize the currently published literature on topics that, in our view, have not yet been sufficiently addressed in the context of HDF. As a narrative review, this work has several limitations that should be acknowledged. We did not follow a predefined protocol, conduct a systematic review with a formal study selection process, perform risk-of-bias assessments, or undertake a quantitative synthesis of the evidence.

Relevant publications were drawn from the authors’ personal libraries and identified through searches of PubMed, supplemented by manual review of the reference lists of key articles, clinical practice guidelines, and previous reviews. Priority was given to randomized controlled trials, large observational studies, registry analyses, meta-analyses, and mechanistic studies relevant to contemporary HDF practice.

Despite substantial efforts to provide a comprehensive cross-section of the available literature, some relevant publications may not have been included. In addition, each study has its own inherent strengths and limitations. Accordingly, the conclusions of this review should be interpreted in the light of the quality, consistency, and evolving nature of the available evidence.

## 7. Conclusions

Overall, available evidence indicates that HDF provides meaningful mechanistic and clinical advantages over HD in the removal of middle-molecular-weight uremic solutes and inflammatory mediators, largely attributable to the addition of convective transport. Consistent with recent meta-analytic evidence including more than 6000 patients, HDF achieves greater reductions in β2M, phosphorus, and CRP, particularly when high convective volumes are delivered, supporting its role in mitigating middle-molecule accumulation and systemic inflammation [162].

However, these benefits should not be interpreted as comprehensive uremic toxin control, as the limited removal of PBUTs remains a fundamental challenge of current extracorporeal therapies. Despite theoretical kinetic advantages of convection, clinical studies have consistently demonstrated only modest or inconsistent improvements in the clearance of key PBUTs such as IS and pCS with HDF compared with HD [52,162]. This limitation reflects the underlying binding thermodynamics governing PBUT–albumin interactions: only the unbound toxin fraction is dialyzable, and convection cannot overcome the strong albumin binding that restricts effective removal [52,162]. In addition, the effectiveness of all dialytic therapies is constrained by compartmental distribution and post-dialytic rebound kinetics; solutes sequestered in intracellular or interstitial compartments redistribute slowly, limiting the net toxin removal achievable during a single session. As discussed in Section 3.4, redistribution and ongoing generation may limit the translation of higher intradialytic clearance into lower interdialytic exposure. We did not implement a compartmental kinetic model; therefore, Figure 1 should be interpreted as conceptual rather than quantitative.

Beyond solute removal, accumulating evidence suggests that HDF may contribute to improved control of chronic micro-inflammation in dialysis patients. Enhanced clearance of pro-inflammatory cytokines and complement factors, together with reduced immune cell activation observed in several clinical studies, indicates that high-volume HDF may attenuate the inflammatory milieu characteristic of ESKD.

From a safety perspective, modern high-volume HDF demonstrates a favorable profile when appropriate technical standards are maintained. Improved hemodynamic stability, fewer intradialytic symptoms, and lower long-term β2M accumulation have been reported, while available evidence has not identified a consistent increase in major device-related or infectious complications compared with HD.

The survival, inflammatory, and hemodynamic benefits discussed in this review are most consistently demonstrated at post-dilution convection volumes of approximately 23 L/session or higher; benefits at lower volumes are less consistent and, in some trials, absent at the primary-endpoint level. Whether centers unable to reliably deliver these convection volumes can expect proportional benefits from high-volume HDF remains an open question, and direct extrapolation of the findings summarized here to such settings is not warranted.

Future progress in dialysis therapy may lie in strategies specifically designed to overcome protein-binding constraints. Emerging approaches such as albumin-binding displacement, adsorption-based technologies, and novel membrane designs aim to increase the free fraction in or directly capture PBUTs from the extracorporeal circuit. Integrating targeted PBUT removal with individualized patient selection for optimized high-volume HDF may represent a promising path toward more comprehensive uremic toxin control and improved long-term outcomes for patients with ESKD.

## Data Availability

No new data were created or analyzed in this study.

## Abbreviations

The following abbreviations are used in this manuscript:

β2M	β2-microglobulin
CRP	C-reactive protein
ESKD	End-stage kidney disease
FLC	Free light chains
HD	Hemodialysis
HDF	Hemodiafiltration
HDx	Expanded hemodialysis
IDH	Intradialytic Hypotension
IL	Interleukin
IS	Indoxyl sulfate
MCO	Medium cut-off
PBUT	Protein-Bound Uremic Toxin
pCS	p-Cresyl sulfate
QoL	Quality of Life
TNF-α	Tumor necrosis factor α

## References

[1] Maduell F., Rodriguez-Espinosa D., Broseta J.J. (2024). Latest Trends in Hemodiafiltration. J. Clin. Med..

[2] Kusirisin P., Srisawat N. (2022). Hemodiafiltration in developing countries. Semin. Dial..

[3] Blankestijn P.J., Vernooij R.W.M., Hockham C., Strippoli G.F.M., Canaud B., Hegbrant J., Barth C., Covic A., Cromm K., Cucui A. (2023). Effect of Hemodiafiltration or Hemodialysis on Mortality in Kidney Failure. N. Engl. J. Med..

[4] Zhang Y., Winter A., Ferreras B.A., Carioni P., Arkossy O., Anger M., Kossmann R., Usvyat L.A., Stuard S., Maddux F.W. (2025). Real-world effectiveness of hemodialysis modalities: A retrospective cohort study. BMC Nephrol..

[5] Zhang Y., Winter A., Ficociello L.H., Arya S., Stuard S., Usvyat L.A., Kalantar-Zadeh K. (2026). Hemodialysis Modality and Mortality Outcomes among Incident Dialysis Patients: An International Cohort Study Comparing High-Volume Hemodiafiltration and Hemodialysis. Clin. J. Am. Soc. Nephrol..

[6] Spiegel S.M., Tressler D., Ficociello L., van Zandt C.R., Anger M.S., Chatoth D.K., Usvyat L.A., Hippen B.E., Stuard S. (2025). Implementation of Online High-Volume Hemodiafiltration in a Chronic Hemodialysis Center in the United States. J. Am. Soc. Nephrol..

[7] Maduell F., Moreso F., Pons M., Ramos R., Mora-Macia J., Carreras J., Soler J., Torres F., Campistol J.M., Martinez-Castelao A. (2013). High-efficiency postdilution online hemodiafiltration reduces all-cause mortality in hemodialysis patients. J. Am. Soc. Nephrol..

[8] Guimaraes M.G.M., Tapioca F.P.M., Dos Santos N.R., Ferreira F.P.d.C.T., Passos L.C.S., Rocha P.N. (2024). Hemodiafiltration versus Hemodialysis in End-Stage Kidney Disease: A Systematic Review and Meta-Analysis. Kidney Med..

[9] Vernooij R.W.M., Hockham C., Strippoli G., Green S., Hegbrant J., Davenport A., Barth C., Canaud B., Woodward M., Blankestijn P.J. (2024). Haemodiafiltration versus haemodialysis for kidney failure: An individual patient data meta-analysis of randomised controlled trials. Lancet.

[10] Peters S.A., Bots M.L., Canaud B., Davenport A., Grooteman M.P., Kircelli F., Locatelli F., Maduell F., Morena M., Nube M.J. (2016). Haemodiafiltration and mortality in end-stage kidney disease patients: A pooled individual participant data analysis from four randomized controlled trials. Nephrol. Dial. Transplant..

[11] Mercadal L., Franck J.E., Metzger M., Torres P.U., de Cornelissen F., Edet S., Bechade C., Vigneau C., Drueke T., Jacquelinet C. (2016). Hemodiafiltration Versus Hemodialysis and Survival in Patients with ESRD: The French Renal Epidemiology and Information Network (REIN) Registry. Am. J. Kidney Dis..

[12] See E.J., Hedley J., Agar J.W.M., Hawley C.M., Johnson D.W., Kelly P.J., Lee V.W., Mac K., Polkinghorne K.R., Rabindranath K.S. (2019). Patient survival on haemodiafiltration and haemodialysis: A cohort study using the Australia and New Zealand Dialysis and Transplant Registry. Nephrol. Dial. Transplant..

[13] Kikuchi K., Hamano T., Wada A., Nakai S., Masakane I. (2019). Predilution online hemodiafiltration is associated with improved survival compared with hemodialysis. Kidney Int..

[14] Canaud B., Gagel A., Peters A., Maierhofer A., Stuard S. (2024). Does online high-volume hemodiafiltration offer greater efficiency and sustainability compared with high-flux hemodialysis? A detailed simulation analysis anchored in real-world data. Clin. Kidney J..

[15] Kazes I., Solignac J., Lassalle M., Mercadal L., Couchoud C. (2024). Twenty years of the French Renal Epidemiology and Information Network. Clin. Kidney J..

[16] Garbelli M., Ion Titapiccolo J., Bellocchio F., Stuard S., Brancaccio D., Neri L. (2022). Prolonged patient survival after implementation of a continuous quality improvement programme empowered by digital transformation in a large dialysis network. Nephrol. Dial. Transplant..

[17] Marcelli D., Kirchgessner J., Amato C., Steil H., Mitteregger A., Moscardo V., Carioni C., Orlandini G., Gatti E. (2001). EuCliD (European Clinical Database): A database comparing different realities. J. Nephrol..

[18] Strippoli G.F.M., Cromm K., Pezoulas V.C., Tachos N., Fotiadis D.I., Jaure A., Malyszko J., Anger M., Fischer F., Hegbrant J. (2026). Hemodiafiltration beyond the CONVINCE trial. Clin. Kidney J..

[19] Battaglia Y., Shroff R., Meijers B., Nistor I., Alfano G., Franssen C., Luyckx V., Liakopoulos V., Mantovani A., Baciga F. (2025). Haemodiafiltration versus high-flux haemodialysis-a Consensus Statement from the EuDial Working Group of the ERA. Nephrol. Dial. Transplant..

[20] Meijers B., Vega A., Juillard L., Kawanishi H., Kirsch A.H., Maduell F., Massy Z.A., Mitra S., Vanholder R., Ronco C. (2024). Extracorporeal Techniques in Kidney Failure. Blood Purif..

[21] Ward R.A. (2022). Basic prerequisites for on-line, high-volume hemodiafiltration. Semin. Dial..

[22] Pstras L., Ronco C., Tattersall J. (2022). Basic physics of hemodiafiltration. Semin. Dial..

[23] Vanholder R., De Smet R., Glorieux G., Argiles A., Baurmeister U., Brunet P., Clark W., Cohen G., De Deyn P.P., Deppisch R. (2003). Review on uremic toxins: Classification, concentration, and interindividual variability. Kidney Int..

[24] Ledebo I., Blankestijn P.J. (2010). Haemodiafiltration-optimal efficiency and safety. NDT Plus.

[25] Daugirdas J.T. (2022). Comparison of measured vs kinetic-model predicted phosphate removal during hemodialysis and hemodiafiltration. Nephrol. Dial. Transplant..

[26] Ward R.A., Daugirdas J.T. (2024). Kinetics of β-2-Microglobulin with Hemodiafiltration and High-Flux Hemodialysis. Clin. J. Am. Soc. Nephrol..

[27] Canaud B., Davenport A., Morena-Carrere M., Amico M., Molinari N., Cristol J.P. (2025). Continuous beta-2 microglobulin-based clearance highlights superiority of high-Dose HDF over high-flux HD in predicting outcomes. Sci. Rep..

[28] Maduell F., Escudero-Saiz V.J., Cuadrado-Payan E., Rodriguez-Garcia M., Rodas L.M., Fontsere N., Salgado M.D.C., Casals G., Rico N., Broseta J.J. (2025). Comparing hemodialysis and hemodiafiltration performance with and without hemoadsorption. Clin. Kidney J..

[29] Bourguignon C., Chenine L., Bargnoux A.S., Leray-Moragues H., Canaud B., Cristol J.P., Morena M. (2016). Hemodiafiltration improves free light chain removal and normalizes κ/λ ratio in hemodialysis patients. J. Nephrol..

[30] Stuard S., Maddux F.W., Canaud B. (2025). Why High-Volume Post-Dilution Hemodiafiltration Should Be the New Standard in Dialysis Care: A Comprehensive Review of Clinical Outcomes and Mechanisms. J. Clin. Med..

[31] Belmouaz M., Bauwens M., Hauet T., Bossard V., Jamet P., Joly F., Chikhi E., Joffrion S., Gand E., Bridoux F. (2020). Comparison of the removal of uraemic toxins with medium cut-off and high-flux dialysers: A randomized clinical trial. Nephrol. Dial. Transplant..

[32] Maduell F., Broseta J.J., Rodriguez-Espinosa D., Del Risco J., Rodas L.M., Arias-Guillen M., Vera M., Fontsere N., Salgado M.D.C., Rico N. (2022). Comparison of four medium cut-off dialyzers. Clin. Kidney J..

[33] Belmouaz M., Diolez J., Bauwens M., Duthe F., Ecotiere L., Desport E., Bridoux F. (2018). Comparison of hemodialysis with medium cut-off dialyzer and on-line hemodiafiltration on the removal of small and middle-sized molecules. Clin. Nephrol..

[34] Lindgren A., Fjellstedt E., Christensson A. (2020). Comparison of Hemodialysis Using a Medium Cutoff Dialyzer versus Hemodiafiltration: A Controlled Cross-Over Study. Int. J. Nephrol. Renov. Dis..

[35] Biedunkiewicz J., Zakrzewska A., Malgorzewicz S., Komorniczak M., Jasiulewicz K., Plonka N., Tarasewicz A., Jankowska M., Biedunkiewicz B., Debska-Slizien A. (2024). Comparison of uremic toxin removal between expanded hemodialysis and high volume online hemodiafiltrations in different modes. Acta Biochim. Pol..

[36] Lukkanalikitkul E., Kidkaem H., Phonrat M., Prathompong P., Anutrakulchai S. (2025). A randomized trial comparing medium cut-off membrane dialyzers with online hemodiafiltration for uremic toxins clearance in hemodialysis patients. Sci. Rep..

[37] Bergling K., Blankestijn P.J. (2026). Beyond Diffusion: Clinical Perspectives on Online Hemodiafiltration and Medium Cut-Off Dialyzers. Am. J. Kidney Dis..

[38] Meijers B.K., Evenepoel P. (2011). The gut-kidney axis: Indoxyl sulfate, p-cresyl sulfate and CKD progression. Nephrol. Dial. Transplant..

[39] Jansen J., Jankowski J., Gajjala P.R., Wetzels J.F.M., Masereeuw R. (2017). Disposition and clinical implications of protein-bound uremic toxins. Clin. Sci..

[40] van Gelder M.K., Middel I.R., Vernooij R.W.M., Bots M.L., Verhaar M.C., Masereeuw R., Grooteman M.P., Nube M.J., van den Dorpel M.A., Blankestijn P.J. (2020). Protein-Bound Uremic Toxins in Hemodialysis Patients Relate to Residual Kidney Function, Are Not Influenced by Convective Transport, and Do Not Relate to Outcome. Toxins.

[41] Faria M., de Pinho M.N. (2021). Challenges of reducing protein-bound uremic toxin levels in chronic kidney disease and end stage renal disease. Transl. Res..

[42] Duval-Sabatier A., Burtey S., Pelletier M., Laforet M., Dou L., Sallee M., Lorec A.M., Knidiri H., Darbon F., Berland Y. (2023). Systematic Comparison of Uremic Toxin Removal Using Different Hemodialysis Modes: A Single-Center Crossover Prospective Observational Study. Biomedicines.

[43] Lima J.D., Guedes M., Rodrigues S.D., Florido A.C.S., Moreno-Amaral A.N., Barra A.B., Canziani M.E., Cuvello-Neto A., Poli-de-Figueiredo C.E., Pecoits-Filho R. (2022). High-volume hemodiafiltration decreases the pre-dialysis concentrations of indoxyl sulfate and p-cresyl sulfate compared to hemodialysis: A post-hoc analysis from the HDFit randomized controlled trial. J. Nephrol..

[44] Leblanc M., Charbonneau R., Lalumiere G., Cartier P., Deziel C. (1996). Postdialysis urea rebound: Determinants and influence on dialysis delivery in chronic hemodialysis patients. Am. J. Kidney Dis..

[45] Tattersall J.E., Chamney P., Aldridge C., Greenwood R.N. (1996). Recirculation and the post-dialysis rebound. Nephrol. Dial. Transplant..

[46] Al-Wakeel J.S. (1998). Post-dialysis Solutes Rebound: Comparison of Two Protocols for Hemodialysis. Saudi J. Kidney Dis. Transpl..

[47] Jean G., Charra B., Chazot C., Laurent G. (1999). Quest for postdialysis urea rebound-equilibrated Kt/V with only intradialytic urea samples. Kidney Int..

[48] Daugirdas J.T., Greene T., Depner T.A., Leypoldt J., Gotch F., Schulman G. (2004). Factors that affect postdialysis rebound in serum urea concentration, including the rate of dialysis: Results from the HEMO Study. J. Am. Soc. Nephrol..

[49] Haas T., Hillion D., Dongradi G. (1991). Phosphate kinetics in dialysis patients. Nephrol. Dial. Transplant..

[50] Pohlmeier R., Vienken J. (2001). Phosphate removal and hemodialysis conditions. Kidney Int..

[51] Stremke E.R., Trevino L., Doshi S., Moorthi R.N., Hill Gallant K.M., Moe S.M. (2022). Postdialysis serum phosphate equilibrium in hemodialysis patients on a controlled diet and no binders. Hemodial. Int..

[52] de Vries J.C., Bras J.G., de Vries G.M., Vollenbroek J.C., Wieringa F.P., Jankowski J., Verhaar M.C., Stamatialis D., Masereeuw R., Gerritsen K.G.F. (2026). Strategies for Removal of Protein-Bound Uremic Toxins in Hemodialysis. Toxins.

[53] de Jager D.J., Grootendorst D.C., Jager K.J., van Dijk P.C., Tomas L.M.J., Ansell D., Collart F., Finne P., Heaf J.G., De Meester J. (2009). Cardiovascular and noncardiovascular mortality among patients starting dialysis. JAMA.

[54] Foley R.N., Parfrey P.S., Sarnak M.J. (1998). Clinical epidemiology of cardiovascular disease in chronic renal disease. Am. J. Kidney Dis..

[55] Go A.S., Chertow G.M., Fan D., McCulloch C.E., Hsu C.Y. (2004). Chronic kidney disease and the risks of death, cardiovascular events, and hospitalization. N. Engl. J. Med..

[56] Petho A.G., Fulop T., Orosz P., Szenasi G., Tapolyai M., Dezsi L. (2025). Increased Cardiovascular Mortality in Hemodialysis: The Role of Chronic Inflammation, Complement Activation, and Non-Biocompatibility. Toxins.

[57] Pedreros-Rosales C., Jara A., Lorca E., Mezzano S., Pecoits-Filho R., Herrera P. (2023). Unveiling the Clinical Benefits of High-Volume Hemodiafiltration: Optimizing the Removal of Medium-Weight Uremic Toxins and Beyond. Toxins.

[58] den Hoedt C.H., Bots M.L., Grooteman M.P., van der Weerd N.C., Mazairac A.H., Penne E.L., Levesque R., ter Wee P.M., Nube M.J., Blankestijn P.J. (2014). Online hemodiafiltration reduces systemic inflammation compared to low-flux hemodialysis. Kidney Int..

[59] Panichi V., Rizza G.M., Paoletti S., Bigazzi R., Aloisi M., Barsotti G., Rindi P., Donati G., Antonelli A., Panicucci E. (2008). Chronic inflammation and mortality in haemodialysis: Effect of different renal replacement therapies. Results from the RISCAVID study. Nephrol. Dial. Transplant..

[60] Agbas A., Canpolat N., Caliskan S., Yilmaz A., Ekmekci H., Mayes M., Aitkenhead H., Schaefer F., Sever L., Shroff R. (2018). Hemodiafiltration is associated with reduced inflammation, oxidative stress and improved endothelial risk profile compared to high-flux hemodialysis in children. PLoS ONE.

[61] Filiopoulos V., Hadjiyannakos D., Metaxaki P., Sideris V., Takouli L., Anogiati A., Vlassopoulos D. (2008). Inflammation and oxidative stress in patients on hemodiafiltration. Am. J. Nephrol..

[62] Fischer D.C., Smith C., De Zan F., Bacchetta J., Bakkaloglu S.A., Agbas A., Anarat A., Aoun B., Askiti V., Azukaitis K. (2021). Hemodiafiltration Is Associated with Reduced Inflammation and Increased Bone Formation Compared with Conventional Hemodialysis in Children: The HDF, Hearts and Heights (3H) Study. Kidney Int. Rep..

[63] Kuo H.L., Chou C.Y., Liu Y.L., Yang Y.F., Huang C.C., Lin H.H. (2008). Reduction of pro-inflammatory cytokines through hemodiafiltration. Ren. Fail..

[64] Bowry S.K., Kircelli F., Himmele R., Nigwekar S.U. (2021). Blood-incompatibility in haemodialysis: Alleviating inflammation and effects of coagulation. Clin. Kidney J..

[65] Campo S., Lacquaniti A., Trombetta D., Smeriglio A., Monardo P. (2022). Immune System Dysfunction and Inflammation in Hemodialysis Patients: Two Sides of the Same Coin. J. Clin. Med..

[66] Ekdahl K.N., Soveri I., Hilborn J., Fellstrom B., Nilsson B. (2017). Cardiovascular disease in haemodialysis: Role of the intravascular innate immune system. Nat. Rev. Nephrol..

[67] Losappio V., Franzin R., Infante B., Godeas G., Gesualdo L., Fersini A., Castellano G., Stallone G. (2020). Molecular Mechanisms of Premature Aging in Hemodialysis: The Complex Interplay Between Innate and Adaptive Immune Dysfunction. Int. J. Mol. Sci..

[68] Nilsson B., Ekdahl K.N., Mollnes T.E., Lambris J.D. (2007). The role of complement in biomaterial-induced inflammation. Mol. Immunol..

[69] Poppelaars F., Faria B., Gaya da Costa M., Franssen C.F.M., van Son W.J., Berger S.P., Daha M.R., Seelen M.A. (2018). The Complement System in Dialysis: A Forgotten Story?. Front. Immunol..

[70] Ward R.A., Schmidt B., Hullin J., Hillebrand G.F., Samtleben W. (2000). A comparison of on-line hemodiafiltration and high-flux hemodialysis: A prospective clinical study. J. Am. Soc. Nephrol..

[71] Elsayed H., Elsharkawy M., Abd ElWahab S., Tawfik A., Fayez M., Khedr L. (2024). Effect of High Dose Hemodiafiltration on Adiponectin and Complement Factor D Removal. Egypt. J. Hosp. Med..

[72] Kato S., Chmielewski M., Honda H., Pecoits-Filho R., Matsuo S., Yuzawa Y., Tranaeus A., Stenvinkel P., Lindholm B. (2008). Aspects of immune dysfunction in end-stage renal disease. Clin. J. Am. Soc. Nephrol..

[73] Rogacev K.S., Ziegelin M., Ulrich C., Seiler S., Girndt M., Fliser D., Heine G.H. (2009). Haemodialysis-induced transient CD16^+^ monocytopenia and cardiovascular outcome. Nephrol. Dial. Transplant..

[74] Sester U., Sester M., Heine G., Kaul H., Girndt M., Kohler H. (2001). Strong depletion of CD14^+^CD16^+^ monocytes during haemodialysis treatment. Nephrol. Dial. Transplant..

[75] Heine G.H., Ulrich C., Seibert E., Seiler S., Marell J., Reichart B., Krause M., Schlitt A., Kohler H., Girndt M. (2008). CD14^++^CD16^+^ monocytes but not total monocyte numbers predict cardiovascular events in dialysis patients. Kidney Int..

[76] Cros J., Cagnard N., Woollard K., Patey N., Zhang S.Y., Senechal B., Puel A., Biswas S.K., Moshous D., Picard C. (2010). Human CD14dim monocytes patrol and sense nucleic acids and viruses via TLR7 and TLR8 receptors. Immunity.

[77] Zawada A.M., Rogacev K.S., Rotter B., Winter P., Marell R.R., Fliser D., Heine G.H. (2011). SuperSAGE evidence for CD14^++^CD16^+^ monocytes as a third monocyte subset. Blood.

[78] Zawada A.M., Schneider J.S., Michel A.I., Rogacev K.S., Hummel B., Krezdorn N., Muller S., Rotter B., Winter P., Obeid R. (2016). DNA methylation profiling reveals differences in the 3 human monocyte subsets and identifies uremia to induce DNA methylation changes during differentiation. Epigenetics.

[79] Carracedo J., Merino A., Nogueras S., Carretero D., Berdud I., Ramirez R., Tetta C., Rodríguez M., Martin-Malo A., Aljama P. (2006). On-line hemodiafiltration reduces the proinflammatory CD14^+^CD16^+^ monocyte-derived dendritic cells: A prospective, crossover study. J. Am. Soc. Nephrol..

[80] Ramirez R., Carracedo J., Merino A., Nogueras S., Alvarez-Lara M.A., Rodriguez M., Martin-Malo A., Tetta C., Aljama P. (2007). Microinflammation induces endothelial damage in hemodialysis patients: The role of convective transport. Kidney Int..

[81] Rama I., Llaudo I., Fontova P., Cerezo G., Soto C., Javierre C., Hueso M., Montero N., Martinez-Castelao A., Torras J. (2016). Online Haemodiafiltration Improves Inflammatory State in Dialysis Patients: A Longitudinal Study. PLoS ONE.

[82] Davenport A. (2023). Why is Intradialytic Hypotension the Commonest Complication of Outpatient Dialysis Treatments?. Kidney Int. Rep..

[83] Burton J.O., Jefferies H.J., Selby N.M., McIntyre C.W. (2009). Hemodialysis-induced repetitive myocardial injury results in global and segmental reduction in systolic cardiac function. Clin. J. Am. Soc. Nephrol..

[84] Flythe J.E., Xue H., Lynch K.E., Curhan G.C., Brunelli S.M. (2015). Association of mortality risk with various definitions of intradialytic hypotension. J. Am. Soc. Nephrol..

[85] Sars B., van der Sande F.M., Kooman J.P. (2020). Intradialytic Hypotension: Mechanisms and Outcome. Blood Purif..

[86] Stuard S., Ridel C., Cioffi M., Trost-Rupnik A., Gurevich K., Bojic M., Karibayev Y., Mohebbi N., Marcinkowski W., Kupres V. (2024). Hemodialysis Procedures for Stable Incident and Prevalent Patients Optimize Hemodynamic Stability, Dialysis Dose, Electrolytes, and Fluid Balance. J. Clin. Med..

[87] Locatelli F., Altieri P., Andrulli S., Bolasco P., Sau G., Pedrini L.A., Basile C., David S., Feriani M., Montagna G. (2010). Hemofiltration and hemodiafiltration reduce intradialytic hypotension in ESRD. J. Am. Soc. Nephrol..

[88] Rootjes P.A., Chaara S., van Zuijdewijn C.L.M.d.R., Nube M.J., Wijngaarden G., Grooteman M.P.C. (2022). High-Volume Hemodiafiltration and Cool Hemodialysis Have a Beneficial Effect on Intradialytic Hemodynamics: A Randomized Cross-Over Trial of Four Intermittent Dialysis Strategies. Kidney Int. Rep..

[89] Morena M., Jaussent A., Chalabi L., Leray-Moragues H., Chenine L., Debure A., Thibaudin D., Azzouz L., Patrier L., Maurice F. (2017). Treatment tolerance and patient-reported outcomes favor online hemodiafiltration compared to high-flux hemodialysis in the elderly. Kidney Int..

[90] Van Der Sande F.M., Kooman J.P., Konings C.J., Leunissen K.M.L. (2001). Thermal effects and blood pressure response during postdilution hemodiafiltration and hemodialysis: The effect of amount of replacement fluid and dialysate temperature. J. Am. Soc. Nephrol..

[91] Donauer J., Schweiger C., Rumberger B., Krumme B., Bohler J. (2003). Reduction of hypotensive side effects during online-haemodiafiltration and low temperature haemodialysis. Nephrol. Dial. Transplant..

[92] Wang A.Y., Ninomiya T., Al-Kahwa A., Perkovic V., Gallagher M.P., Hawley C., Jardine M.J. (2014). Effect of hemodiafiltration or hemofiltration compared with hemodialysis on mortality and cardiovascular disease in chronic kidney failure: A systematic review and meta-analysis of randomized trials. Am. J. Kidney Dis..

[93] Maggiore Q., Pizzarelli F., Sisca S., Zoccali C., Parlongo S., Nicolo F., Creazzo G. (1982). Blood temperature and vascular stability during hemodialysis and hemofiltration. Trans. Am. Soc. Artif. Intern. Organs.

[94] Canzanello V.J., Burkart J.M. (1992). Hemodialysis-Associated Muscle Cramps. Semin. Dial..

[95] Chillar R.K., Desforges J.F. (1972). Muscular cramps during maintenance haemodialysis. Lancet.

[96] Punj S., Enaam A., Marquez A., Atkinson A.J., Batlle D. (2020). A Survey on Dialysis-Related Muscle Cramping and a Hypothesis of Angiotensin II on Its Pathophysiology. Kidney Int. Rep..

[97] Sav M.Y., Sav T., Senocak E., Sav N.M. (2014). Hemodialysis-related headache. Hemodial. Int..

[98] Scherer J.S., Combs S.A., Brennan F. (2017). Sleep Disorders, Restless Legs Syndrome, and Uremic Pruritus: Diagnosis and Treatment of Common Symptoms in Dialysis Patients. Am. J. Kidney Dis..

[99] Rose M., Fischer F.H., Liegl G., Strippoli G.F.M., Hockham C., Vernooij R.W.M., Barth C., Canaud B., Covic A., Cromm K. (2024). The CONVINCE randomized trial found positive effects on quality of life for patients with chronic kidney disease treated with hemodiafiltration. Kidney Int..

[100] Karkar A., Abdelrahman M., Locatelli F. (2015). A Randomized Trial on Health-Related Patient Satisfaction Level with High-Efficiency Online Hemodiafiltration versus High-Flux Dialysis. Blood Purif..

[101] Chen Z.J., Cao G., Tang W.X., Lv X.Y., Huang S.M., Qin W., Ping F., Ye T. (2009). A randomized controlled trial of high-permeability haemodialysis against conventional haemodialysis in the treatment of uraemic pruritus. Clin. Exp. Dermatol..

[102] Hazim A., Adarmouch L., Eloury A., Aasfara J., Asly M., Slassi I. (2021). Hemodialysis-related headache: Still a challenge in 2020? Effect of conventional versus online hemodiafiltration from a study in Casablanca, Morocco. Artif. Organs.

[103] Jiang X., Ji F., Chen Z.W., Huang Q.L. (2016). Comparison of high-flux hemodialysis with hemodialysis filtration in treatment of uraemic pruritus: A randomized controlled trial. Int. Urol. Nephrol..

[104] Penne E.L., van der Weerd N.C., van den Dorpel M.A., Grooteman M.P., Levesque R., Nube M.J., Bots M.L., Blankestijn P.J., ter Wee P.M., CONTRAST Investigators (2010). Short-term effects of online hemodiafiltration on phosphate control: A result from the randomized controlled Convective Transport Study (CONTRAST). Am. J. Kidney Dis..

[105] Davenport A., Gardner C., Delaney M., Pan Thames Renal Audit Group (2010). The effect of dialysis modality on phosphate control: Haemodialysis compared to haemodiafiltration. The Pan Thames Renal Audit. Nephrol. Dial. Transplant..

[106] Movilli E., Camerini C., Gaggia P., Poiatti P., Pola A., Viola B.F., Zubani R., Jeannin G., Cancarini G. (2011). Effect of post-dilutional on-line haemodiafiltration on serum calcium, phosphate and parathyroid hormone concentrations in uraemic patients. Nephrol. Dial. Transplant..

[107] Karaboyas A., Zee J., Brunelli S.M., Usvyat L.A., Weiner D.E., Maddux F.W., Nissenson A.R., Jadoul M., Locatelli F., Winkelmayer W.C. (2017). Dialysate Potassium, Serum Potassium, Mortality, and Arrhythmia Events in Hemodialysis: Results from the Dialysis Outcomes and Practice Patterns Study (DOPPS). Am. J. Kidney Dis..

[108] Grooteman M.P., van den Dorpel M.A., Bots M.L., Penne E.L., van der Weerd N.C., Mazairac A.H., den Hoedt C.H., van der Tweel I., Levesque R., Nube M.J. (2012). Effect of online hemodiafiltration on all-cause mortality and cardiovascular outcomes. J. Am. Soc. Nephrol..

[109] Ok E., Asci G., Toz H., Ok E.S., Kircelli F., Yilmaz M., Hur E., Demirci M.S., Demirci C., Duman S. (2013). Mortality and cardiovascular events in online haemodiafiltration (OL-HDF) compared with high-flux dialysis: Results from the Turkish OL-HDF Study. Nephrol. Dial. Transplant..

[110] Locatelli F., Marcelli D., Conte F., Limido A., Malberti F., Spotti D. (1999). Comparison of mortality in ESRD patients on convective and diffusive extracorporeal treatments. Kidney Int..

[111] Koda Y., Nishi S.I., Miyazaki S., Haginoshita S., Sakurabayashi T., Suzuki M., Sakai S., Yuasa Y., Hirasawa Y., Nishi T. (1997). Switch from conventional to high-flux membrane reduces the risk of carpal tunnel syndrome and mortality of hemodialysis patients. Kidney Int..

[112] Lornoy W., Becaus I., Billiouw J.M., Sierens L., Van Malderen P., D’Haenens P. (2000). On-line haemodiafiltration. Remarkable removal of β2-microglobulin. Long-term clinical observations. Nephrol. Dial. Transplant..

[113] Floege J., Ketteler M. (2001). β2-microglobulin-derived amyloidosis: An update. Kidney Int..

[114] Susantitaphong P., Siribamrungwong M., Jaber B.L. (2013). Convective therapies versus low-flux hemodialysis for chronic kidney failure: A meta-analysis of randomized controlled trials. Nephrol. Dial. Transplant..

[115] Murtas S., Aquilani R., Iadarola P., Deiana M.L., Secci R., Cadeddu M., Bolasco P. (2020). Differences and Effects of Metabolic Fate of Individual Amino Acid Loss in High-Efficiency Hemodialysis and Hemodiafiltration. J. Ren. Nutr..

[116] Bevier A., Novel-Catin E., Blond E., Pelletier S., Parant F., Koppe L., Fouque D. (2022). Water-Soluble Vitamins and Trace Elements Losses during On-Line Hemodiafiltration. Nutrients.

[117] Molina P., Vizcaino B., Molina M.D., Beltran S., Gonzalez-Moya M., Mora A., Castro-Alonso C., Kanter J., Avila A.I., Gorriz J.L. (2018). The effect of high-volume online haemodiafiltration on nutritional status and body composition: The ProtEin Stores prEservaTion (PESET) study. Nephrol. Dial. Transplant..

[118] Tharmaraj D., Kerr P.G. (2017). Haemolysis in haemodialysis. Nephrology.

[119] Greenberg K.I., Choi M.J. (2021). Hemodialysis Emergencies: Core Curriculum 2021. Am. J. Kidney Dis..

[120] Morena M., Creput C., Bouzernidj M., Rodriguez A., Chalabi L., Seigneuric B., Lauret C., Bargnoux A.S., Dupuy A.M., Cristol J.P. (2019). Randomised trial on clinical performances and biocompatibility of four high-flux hemodialyzers in two mode treatments: Hemodialysis vs post dilution hemodiafiltration. Sci. Rep..

[121] Penne E.L., Visser L., van den Dorpel M.A., van der Weerd N.C., Mazairac A.H., van Jaarsveld B.C., Koopman M.G., Vos P., Feith G.W., Hovinga T.K.K. (2009). Microbiological quality and quality control of purified water and ultrapure dialysis fluids for online hemodiafiltration in routine clinical practice. Kidney Int..

[122] Catapano G., Morrone G., Hu L., Fragomeni G., Buscaroli A. (2025). Endotoxin-Retentive Filters for the Online Preparation of Ultrapure Dialysis Fluid and Non-Pyrogenic Substitution Fluid: A Critical Review and Reference Guide. Membranes.

[123] Guha C., Gallego D., Grandinetti A., Warren M., Jaure A. (2023). Patient Perspectives on Clotting in the Extracorporeal Circuit and Decision-Making Regarding Anticoagulation Therapy. Semin. Nephrol..

[124] MacRae J.M., Dipchand C., Oliver M., Moist L., Lok C., Clark E., Hiremath S., Kappel J., Kiaii M., Luscombe R. (2016). Arteriovenous Access Failure, Stenosis, and Thrombosis. Can. J. Kidney Health Dis..

[125] Lok C.E., Yuo T., Lee T. (2025). Hemodialysis Vascular Access: Core Curriculum 2025. Am. J. Kidney Dis..

[126] Pecoits-Filho R., Larkin J., Poli-de-Figueiredo C.E., Cuvello-Neto A.L., Barra A.B.L., Goncalves P.B., Sheth S., Guedes M., Han M., Calice-Silva V. (2021). Effect of hemodiafiltration on measured physical activity: Primary results of the HDFIT randomized controlled trial. Nephrol. Dial. Transplant..

[127] Sanchez Y.P., Romero R.A., Campbell S.M., Thompson S., Bohm C., MacRae J., Davison S.N., Bello A.K., Courtney M., Tangri N. (2025). The Impact of Hemodiafiltration Versus Hemodialysis on Uremic Symptoms, Physical Function: A Systematic Review and Meta-analysis of Randomized Controlled Trials. Kidney Med..

[128] La Milia V., Ravasi C., Carfagna F., Alberghini E., Baragetti I., Buzzi L., Ferrario F., Furiani S., Barbone G.S., Pontoriero G. (2019). Sodium removal and plasma tonicity balance are not different in hemodialysis and hemodiafiltration using high-flux membranes. J. Nephrol..

[129] Jaques D.A., Chhabra R., Khatri P., Davenport A. (2025). Impact of convective clearance on intra-dialytic potassium removal in chronic dialysis patients. Artif. Organs.

[130] Schiffl H. (2007). Prospective randomized cross-over long-term comparison of online haemodiafiltration and ultrapure high-flux haemodialysis. Eur. J. Med. Res..

[131] Locatelli F., Del Vecchio L., Violo L., Pontoriero G. (2014). Phosphate binders for the treatment of hyperphosphatemia in chronic kidney disease patients on dialysis: A comparison of safety profiles. Expert Opin. Drug Saf..

[132] Pun P.H., Middleton J.P. (2017). Dialysate Potassium, Dialysate Magnesium, and Hemodialysis Risk. J. Am. Soc. Nephrol..

[133] Ohtake T., Oka M., Ishioka K., Honda K., Mochida Y., Maesato K., Moriya H., Hidaka S., Kobayashi S. (2012). Cardiovascular protective effects of on-line hemodiafiltration: Comparison with conventional hemodialysis. Ther. Apher. Dial..

[134] Imamovic G., Hrvacevic R., Kapun S., Marcelli D., Bayh I., Grassmann A., Scatizzi L., Maslovaric J., Canaud B. (2014). Survival of incident patients on high-volume online hemodiafiltration compared to low-volume online hemodiafiltration and high-flux hemodialysis. Int. Urol. Nephrol..

[135] da Rocha E.P., Kojima C.A., Modelli de Andrade L.G., Costa D.M., Magalhaes A.O., Rocha W.F., de Vasconcelos L.N., Rosa M.G., Wagner Martins C.S. (2024). Comparing Survival Outcomes between Hemodialysis and Hemodiafiltration Using Real-World Data from Brazil. J. Clin. Med..

[136] Strogoff-de-Matos J.P., Canziani M.E.F., Barra A.B.L. (2025). Mortality on Hemodiafiltration Compared to High-Flux Hemodialysis: A Brazilian Cohort Study. Am. J. Kidney Dis..

[137] Canaud B., Strippoli G., Davenport A. (2025). High-Volume Hemodiafiltration Versus High-Flux Hemodialysis: A Narrative Review for the Clinician. J. Clin. Med..

[138] Stuard S., Maddux F.W. (2025). High-Volume Hemodiafiltration: Expanding the Evidence Beyond Randomized Trials-A Critical Perspective on the 2025 EuDial Consensus. J. Clin. Med..

[139] Gal R., Korzets A., Schwartz A., Rath-Wolfson L., Gafter U. (1994). Systemic distribution of beta 2-microglobulin-derived amyloidosis in patients who undergo long-term hemodialysis. Report of seven cases and review of the literature. Arch. Pathol. Lab. Med..

[140] Takayama F., Miyazaki S., Morita T., Hirasawa Y., Niwa T. (2001). Dialysis-related amyloidosis of the heart in long-term hemodialysis patients. Kidney Int..

[141] Morten I.J., Hewitt E.W., Radford S.E., Uversky V.N., Fink A.L. (2007). β2-Microglobulin and Dialysis-Related Amyloidosis. Protein Misfolding, Aggregation, and Conformational Diseases: Part B: Molecular Mechanisms of Conformational Diseases.

[142] Portales-Castillo I., Yee J., Tanaka H., Fenves A.Z. (2020). Beta-2 Microglobulin Amyloidosis: Past, Present, and Future. Kidney360.

[143] Paglialonga F., Monzani A., Prodam F., Smith C., De Zan F., Canpolat N., Agbas A., Bayazit A., Anarat A., Bakkaloglu S.A. (2023). Nutritional and Anthropometric Indices in Children Receiving Haemodiafiltration vs Conventional Haemodialysis—The HDF, Heart and Height (3H) Study. J. Ren. Nutr..

[144] Macias N., Vega A., Abad S., Santos A., Cedeno S., Linares T., Garcia-Prieto A.M., Aragoncillo I., Yuste C., Lopez-Gomez J.M. (2017). Is High-Volume Online Hemodiafiltration Associated with Malnutrition? *Ther*. Apher. Dial..

[145] Polaschegg H.D. (2009). Red blood cell damage from extracorporeal circulation in hemodialysis. Semin. Dial..

[146] Tattersall J., Martin-Malo A., Pedrini L., Basci A., Canaud B., Fouque D., Haage P., Konner K., Kooman J., Pizzarelli F. (2007). EBPG guideline on dialysis strategies. Nephrol. Dial. Transplant..

[147] Ronco C. (2015). Hemodiafiltration: Technical and Clinical Issues. Blood Purif..

[148] Ni Y., Wu W., Zhou H., Li M., Zhu X., Niu H., Liu J., Xue L., Liu Y., Yang M. (2024). Effect of central dialysis fluid delivery system on markers of inflammation in hemodialysis patients. BMC Nephrol..

[149] Hoen B., Paul-Dauphin A., Hestin D., Kessler M. (1998). EPIBACDIAL: A multicenter prospective study of risk factors for bacteremia in chronic hemodialysis patients. J. Am. Soc. Nephrol..

[150] Roth V.R., Jarvis W.R. (2000). Outbreaks of infection and/or pyrogenic reactions in dialysis patients. Semin. Dial..

[151] Engelen M.M., Verhamme P., Vanassche T. (2023). Clotting of the Extracorporeal Circuit in Hemodialysis: Beyond Contact-Activated Coagulation. Semin. Nephrol..

[152] Fadem S.Z., Fadem S.Z., Moura-Neto J.A., Golper T.A. (2023). Blood Clotting Complications in Dialysis. Complications in Dialysis.

[153] Chiasakul T., Mullier F., Lecompte T., Nguyen P., Cuker A. (2023). Laboratory Monitoring of Heparin Anticoagulation in Hemodialysis: Rationale and Strategies. Semin. Nephrol..

[154] Schild A.F., Perez E., Gillaspie E., Seaver C., Livingstone J., Thibonnier A. (2008). Arteriovenous fistulae vs. arteriovenous grafts: A retrospective review of 1,700 consecutive vascular access cases. J. Vasc. Access.

[155] Sehgal A.R., Grey S.F., DeOreo P.B., Whitehouse P.J. (1997). Prevalence, recognition, and implications of mental impairment among hemodialysis patients. Am. J. Kidney Dis..

[156] Drew D.A., Weiner D.E., Tighiouart H., Duncan S., Gupta A., Scott T., Sarnak M.J. (2017). Cognitive Decline and Its Risk Factors in Prevalent Hemodialysis Patients. Am. J. Kidney Dis..

[157] Tan L.H., Chen P.S., Chiang H.Y., King E., Yeh H.C., Hsiao Y.L., Chang D.R., Chen S.H., Wu M.Y., Kuo C.C. (2022). Insomnia and Poor Sleep in CKD: A Systematic Review and Meta-analysis. Kidney Med..

[158] Murray A.M. (2008). Cognitive impairment in the aging dialysis and chronic kidney disease populations: An occult burden. Adv. Chronic Kidney Dis..

[159] Shroff R., Smith C., Ranchin B., Bayazit A.K., Stefanidis C.J., Askiti V., Azukaitis K., Canpolat N., Agbas A., Aitkenhead H. (2019). Effects of Hemodiafiltration versus Conventional Hemodialysis in Children with ESKD: The HDF, Heart and Height Study. J. Am. Soc. Nephrol..

[160] Ahlmann C., Stronach L., Waters K., Walker K., Oh J., Schmitt C.P., Ranchin B., Shroff R. (2024). Hemodiafiltration for children with stage 5 chronic kidney disease: Technical aspects and outcomes. Pediatr. Nephrol..

[161] Han M., Guedes M., Larkin J., Raimann J.G., Lesqueves Barra A.B., Canziani M.E.F., Cuvello Neto A.L., Poli-de-Figueiredo C.E., Kotanko P., Pecoits-Filho R. (2020). Effect of Hemodiafiltration on Self-Reported Sleep Duration: Results from a Randomized Controlled Trial. Blood Purif..

[162] Wathanavasin W., Jaturapisanukul S., Janwetchasil P., Thongprayoon C., Cheungpasitporn W., Fulop T. (2026). Effects of Hemodiafiltration Versus Hemodialysis on Uremic Toxins, Inflammatory Markers, Anemia, and Nutritional Parameters: A Systematic Review and Meta-Analysis. Toxins.

